# The Seminal Microenvironment as a Potentially Tractable Interface Between Metabolic Disease and Sperm Damage

**DOI:** 10.3390/ijms27219722

**Published:** 2026-10-29

**Authors:** Yunlong Yao, Xi Zhang, Lingru Li, Chenyu Fei, Yunzhi Zhang, Shuo Huang, Yulin Zong, Liujie Zhu, Yaxin Cao, Tianxing Li, Mingyan Shao, Yanfei Zheng

**Affiliations:** Wangqi Academy, National Institute of TCM Constitution and Preventive Medicine, Beijing University of Chinese Medicine, Beijing 100029, China

**Keywords:** seminal microenvironment, metabolic disease, male infertility, oxidative stress, seminal microbiome, sperm DNA fragmentation

## Abstract

Male reproductive disorders are increasingly linked to metabolic diseases, but mechanistic research remains predominantly focused on the testis. The seminal microenvironment, shaped by epididymal and accessory-gland secretions, contains metabolites, lipids, ions, cytokines, extracellular vesicles, and microbial products, and may act as a metabolically responsive post-testicular interface that continues to influence sperm after spermatogenesis. Obesity, insulin resistance, dyslipidemia, and chronic inflammation can remodel this environment by disrupting secretory activity, redox homeostasis, immune signaling, and microbial ecology. These changes may converge on oxidative and inflammatory pathways, promoting membrane lipid peroxidation, mitochondrial dysfunction, impaired motility and capacitation, oxidative DNA base lesions, DNA fragmentation, and potentially epigenetic alterations. Mouse studies separately report obesity-associated epididymal vesicle remodeling and RNA-dependent changes in embryonic gene expression. In this Review, we examine the regulation and metabolic remodeling of the seminal microenvironment, how these changes may translate systemic metabolic stress into sperm injury, and emerging interventions including metabolic correction, antioxidant strategies, and microbiome-targeted approaches. Overall, current evidence remains largely observational and cross-sectional, with additional technical limitations in low-biomass seminal microbiome studies. Longitudinal and mechanistic studies are therefore needed to determine whether the seminal microenvironment is a causal mediator of metabolic infertility and a clinically tractable target for mechanism-guided preconception care.

## 1. Introduction

Over the past four decades, studies across diverse populations have reported declining semen quality [1,2,3,4]. Sperm defects remain important contributors to couple infertility [5,6]. Among modifiable risk factors, obesity and metabolic syndrome are associated with lower semen volume, sperm concentration and motility, abnormal morphology, and reduced vitality [7,8,9,10]. Similar abnormalities have been reported even with high-normal fasting glucose and with increasing body mass index (BMI) [11,12].

The epidemiological association between metabolic disease and impaired male fertility is increasingly being recognized. However, most mechanistic studies of male infertility remain focused on abnormalities in the testes, where sperm are generated through spermatogenesis and spermiogenesis [13]. A recent review highlighted the effects of metabolic disease on testicular metabolism, Sertoli cell function, blood–testis barrier integrity, the hypothalamic–pituitary–gonadal axis, and the epigenome of male germ cells [14]. However, the reproductive journey of spermatozoa does not end when they leave the seminiferous epithelium. Instead, they undergo further maturation in the epididymis, are exposed to secretions from the accessory sex glands during ejaculation, and subsequently traverse the female reproductive tract before fertilization. These post-testicular environments expose sperm to dynamic chemical and immunological conditions that may influence their functional competence and integrity. These conditions can collectively be referred to as the seminal microenvironment. Despite its potential importance, the seminal microenvironment has not been systematically examined. However, there is some precedent for its potential role in male infertility. The “post-testicular sperm environment” has been mentioned in early studies, and the epididymis has been characterized as a “post-testicular compartment” [15,16]. These observations suggest that the seminal microenvironment may serve as an important and potentially modifiable determinant of sperm function beyond the testis.

**Figure 1:**
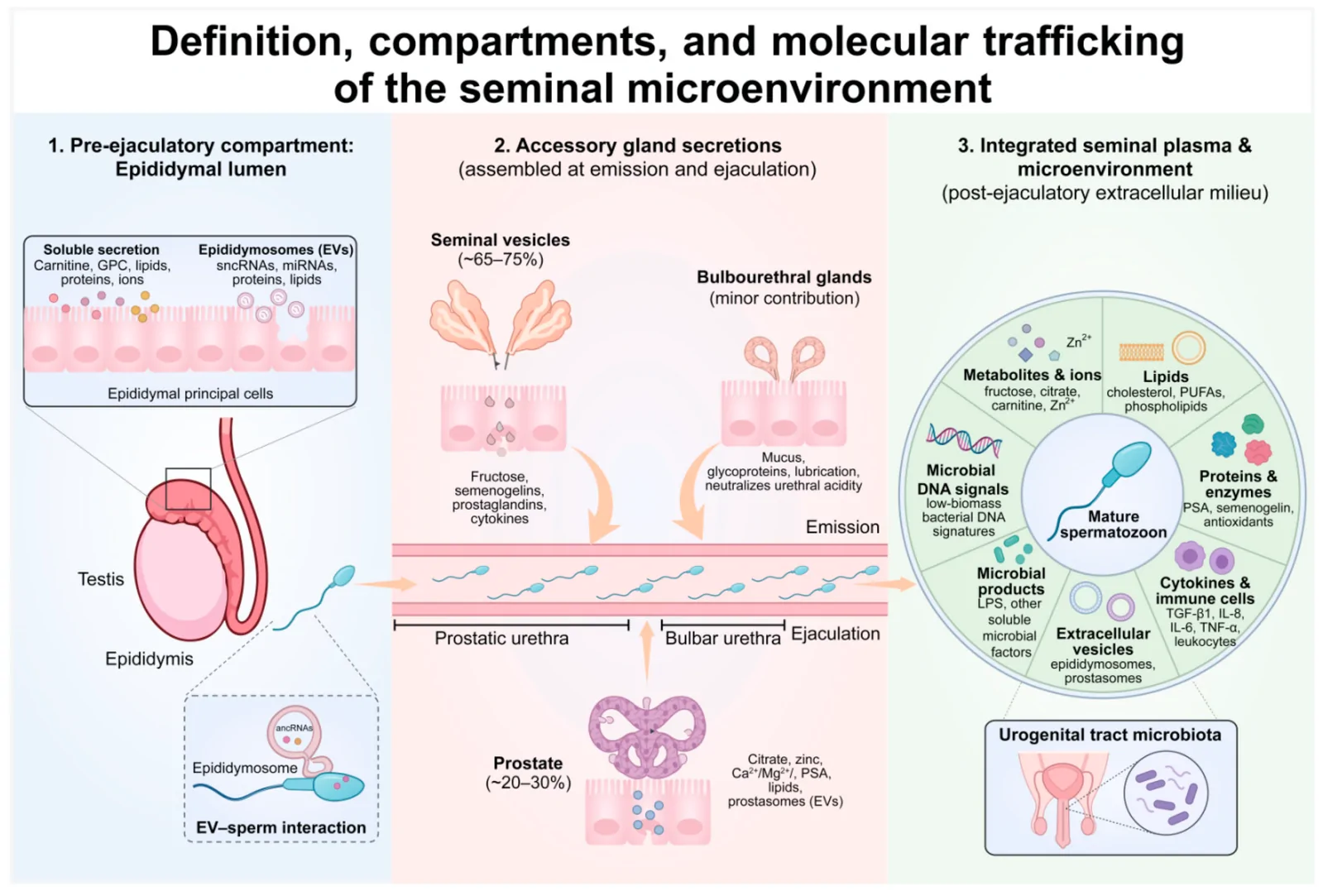
Anatomical compartments and molecular trafficking within the seminal microenvironment. The schematic distinguishes the pre-ejaculatory epididymal lumen, accessory-gland secretions added during emission and ejaculation, and the resulting seminal plasma. During epididymal transit, epithelial cells release soluble factors, including carnitine, glycerophosphocholine, proteins, lipids, and ions, alongside epididymosomes carrying proteins, RNAs, and lipids that can interact with sperm. During emission and ejaculation, the seminal vesicles contribute fructose, semenogelins, prostaglandins, and cytokines; the prostate provides citrate, zinc, enzymes, and extracellular vesicles; and the bulbourethral glands contribute mucus. These secretions form a composite extracellular environment containing metabolites, lipids, proteins, extracellular vesicles, immune mediators, and microbial signals. Microbial DNA signatures and soluble microbial products are illustrated in relation to the urogenital tract. The arrows indicate sperm transport and the addition of secretions across anatomically and temporally distinct compartments.

The seminal microenvironment is defined by its anatomical and biological origins. Seminal plasma is produced by several accessory reproductive glands and accounts for approximately 95% of the final ejaculate volume. The seminal vesicles contribute ~65–75% to seminal plasma; the prostate, ~20–30%; the testes and epididymis, ~2–5%; and the bulbourethral and periurethral glands, ~1% [17,18]. The anatomically and temporally distinct contributions of these compartments are illustrated (Figure 1). Seminal plasma contributes to sperm transport and function. Studies in cattle, pigs, and humans have implicated seminal plasma in the regulation of sperm motility, capacitation, and the acrosome reaction after deposition in the female reproductive tract [18,19,20,21]. Accessory gland secretions are also highly responsive to regulatory physiological and environmental cues. Accumulating evidence shows that environmental stressors can alter the composition and function of seminal plasma by modifying its protein, metabolite, and signaling profiles [22]. The hormonal and metabolic composition of seminal plasma, therefore, has a dual role. That is, it contributes directly to sperm physiology while also serving as a biomarker of systemic health. Hence, seminal plasma can act as a distinctive window into the metabolic–reproductive axis in male infertility [23]. Next-generation sequencing studies show that semen contains a variable microbial community influenced by host and environmental factors [24,25]. Because gut dysbiosis is linked to metabolic disease and male reproductive function, the modest microbial overlap among gut, urine, and semen raises the possibility of cross-compartment interactions [26,27,28]. However, the biological significance and route of such interactions remain unresolved.

In this Review, we examine the seminal microenvironment as a metabolically responsive post-testicular interface. Human observational studies identify associations between metabolic phenotypes, seminal components, and sperm outcomes; experiments on mature sperm and animal reproductive tissues describe selected biological mechanisms. On this basis, we propose a metabolic disease–seminal microenvironment–sperm damage hypothesis and evaluate metabolic, redox, inflammatory, and microbial evidence within this framework.

## 2. Regulation of the Seminal Microenvironment

### 2.1. Accessory Gland Secretions and the Assembly of Seminal Plasma

**Figure 2:**
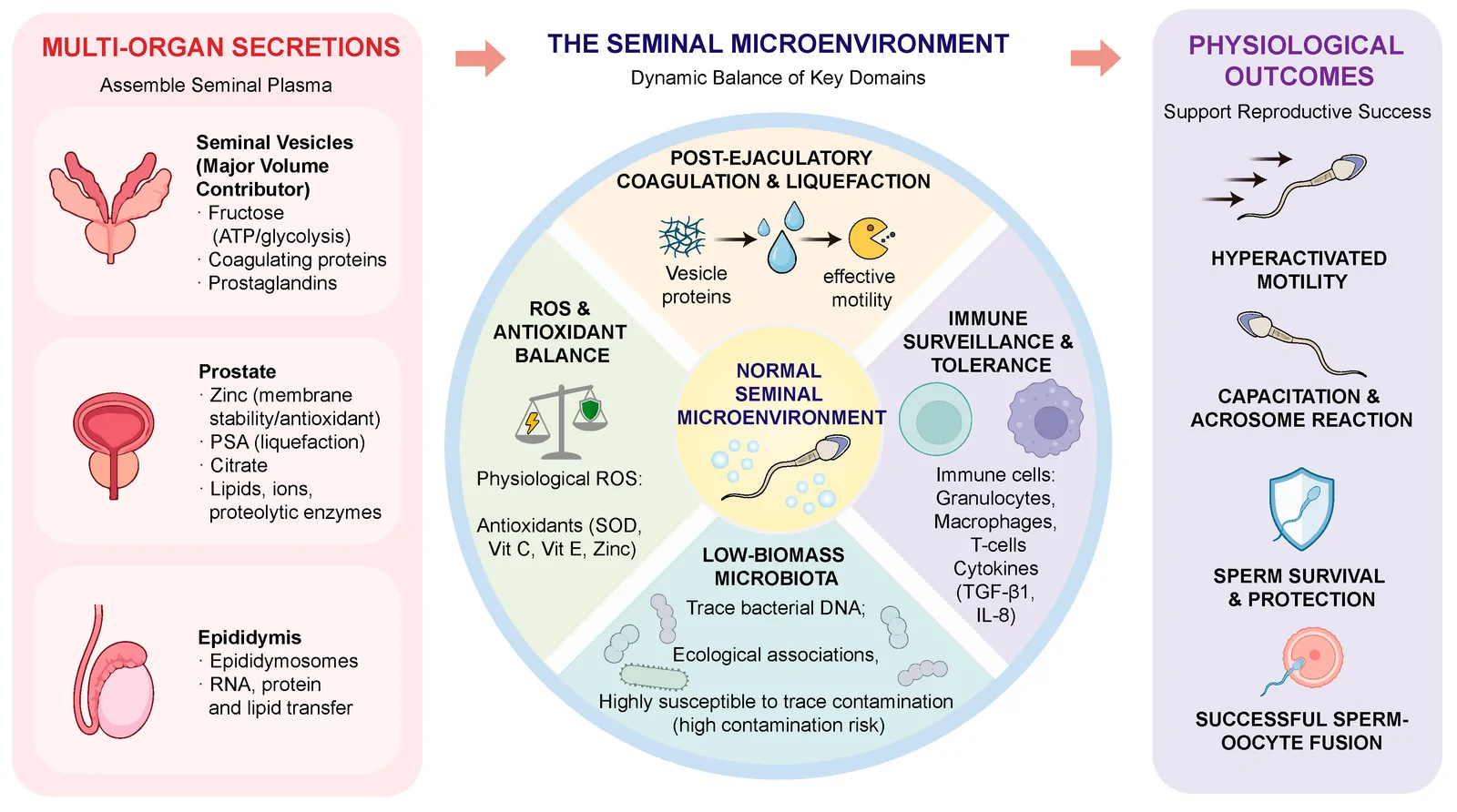
Physiological organization of the normal seminal microenvironment. Seminal plasma is assembled from functionally distinct secretions of the male reproductive tract. The seminal vesicles provide most of the ejaculate volume and contribute fructose, coagulating proteins, prostaglandins, and other factors supporting post-ejaculatory metabolism and coagulation. The prostate contributes zinc, citrate, lipids, ions, proteolytic enzymes, and prostate-specific antigen, which participates in semen liquefaction. The epididymis and testes contribute a smaller volume but provide factors essential for sperm maturation, including epididymosomes carrying proteins, ribonucleic acids, and lipids. Within the resulting seminal microenvironment, sperm are exposed to dynamically regulated coagulation and liquefaction, redox balance between physiological reactive oxygen species and antioxidant defenses, immune surveillance and tolerance, and low-biomass microbial signals that are particularly susceptible to contamination. These components interact rather than functioning as isolated modules. When appropriately balanced, the seminal microenvironment supports sperm survival, hyperactivated motility, capacitation, the acrosome reaction, and ultimately successful sperm–oocyte fusion after ejaculation.

Seminal plasma is produced by several compartments of the male reproductive tract. However, the relative contributions of these compartments vary across different species. In humans, most of the ejaculate volume is derived from the seminal vesicles and prostate. Meanwhile, the epididymis, testes, and bulbourethral glands have a relatively low contribution [29]. However, this volumetric asymmetry does not imply functional asymmetry. Each compartment contributes a distinct set of molecules to the seminal environment. The seminal vesicles provide fructose and prostaglandins; the prostate adds citrate, zinc, and prostatic acid phosphatase; and the epididymis supplies N-acetylglucosaminidase, carnitine, glycerophosphocholine, lipids, carbohydrates, and steroids [30] (Figure 2). Importantly, the epididymal lumen is encountered before ejaculation; secretions from the seminal vesicles and prostate are mixed with sperm predominantly during emission and ejaculation. Concentrations measured in ejaculated plasma therefore do not necessarily represent concentrations experienced during epididymal maturation.

The prostate is a major source of functional constituents in seminal plasma. It secretes a fluid rich in proteolytic enzymes, citrate, lipids, and calcium, magnesium, and zinc ions [31,32,33]. Zinc plays a particularly important role. It serves as a marker of prostatic secretion and contributes to sperm integrity by stabilizing membranes and proteins and limiting oxidative damage [34]. Furthermore, it acts alongside selenium and other antioxidant components to preserve sperm function [35]. Prostate-specific antigen, a serine protease secreted by the prostate, cleaves semenogelins and thereby contributes to liquefaction of the ejaculate and the subsequent increase in sperm motility [36].

The seminal vesicles contribute most of the ejaculate volume and produce secretions rich in semenogelins, fibronectin, prostaglandins, cytokines, and fructose [31,32,33,37]. Fructose acts as an important glycolytic substrate for spermatozoa after ejaculation. Spermatozoa rapidly utilize seminal plasma components to support motility, maintain membrane integrity and prepare for fertilization. Meanwhile, seminal-vesicle-derived fructose enables ATP production under varying oxygen levels [38]. Seminal fructose is also used clinically as a marker of seminal-vesicle function [34].

Although epididymal fluid contributes a small proportion of the final ejaculate volume, it supports sperm maturation during epididymal transit. Epididymal epithelial cells supply soluble proteins, enzymes, ions, and lipids through conventional merocrine secretion, and release extracellular vesicles (EVs) from the apical surface. Epididymosomes carry selected proteins, RNAs, and lipids and can transfer some of these molecules to sperm in experimental systems [39,40,41,42]. The bulbourethral glands produce pre-ejaculatory mucus [43,44]. Together, these secretory processes provide temporally distinct luminal factors during epididymal transit and ejaculation.

Sperm undergo maturation in the epididymal lumen before encountering seminal vesicle, prostatic and bulbourethral secretions during emission and ejaculation [31,32,33,34,35,36,37,38,39,40,41,42]. Semen coagulation and liquefaction subsequently modify the environment surrounding ejaculated sperm. These anatomical and temporal distinctions are important when interpreting measurements made in seminal plasma.

### 2.2. Redox, Immune and Microbial Homeostasis

At physiological concentrations, reactive oxygen species (ROS) support sperm tyrosine phosphorylation, capacitation, hyperactivation, the acrosome reaction and interactions required for fertilization [45,46,47]. Human sperm themselves produce ROS, while enzymatic and non-enzymatic antioxidants in seminal plasma help maintain redox balance [20,48,49,50,51]. These include superoxide dismutase, glutathione peroxidase, catalase, vitamins C and E, selenium, zinc and glutathione. Antioxidant capacity is partly derived from accessory-gland secretions; studies have linked seminal antioxidant-enzyme activities to prostate-associated markers and semen parameters [52,53,54]. One study reported seminal catalase activity of 129 ± 59 nmol O_2_ min^−1^ mL^−1^ in normozoospermic samples [53].

Seminal leukocytes comprise predominantly granulocytes, with smaller macrophage and T-cell populations [55]. The World Health Organization defines leukocytospermia as ≥1 × 10^6^ leukocytes/mL, although elevated counts can occur without confirmed infection or overt inflammation [56,57]. Activated leukocytes can release ROS, chemotactic factors and proteases. Seminal plasma also contains constitutive cytokines, including transforming growth factor-β1 (TGF-β1) and interleukin-8 (IL-8), within a generally tolerogenic immune milieu; these signals may contribute to female reproductive-tract responses after insemination [58,59,60]. In human cross-sectional studies, leukocyte abundance has been associated with semen volume, concentration or motility in some cohorts, while other studies found weaker or inconsistent associations [55,57].

Next-generation sequencing has detected variable microbial DNA signatures in human semen [61]. Interindividual variation is substantial, and samples may show dominance by one bacterial genus rather than a uniform community. Early studies detected taxa including *Ralstonia*, *Corynebacterium* and *Lactobacillus* [62]. In a cohort comparing men with infertility, recurrent pregnancy loss or proven fertility, *Streptococcus*-, *Prevotella*- and *Lactobacillus*/*Gardnerella*-dominant groups were described without distinct species-level clusters [63]. These measurements add a microbial dimension to the secretory, immune and redox conditions experienced by ejaculated sperm.

Understanding the origins of the seminal microbiome is essential for interpreting its potential biological significance. The seminal microbiome is derived from the microbiomes of multiple compartments within the male reproductive tract, including the testes, epididymis, prostate, seminal vesicles, and urethra [64]. Indeed, human testicular tissue itself harbors low-level bacterial DNA signatures. For instance, Alfano et al. detected such signatures in normozoospermic control testes and reported altered profiles in cases of idiopathic non-obstructive azoospermia [65]. Interestingly, vasectomy provides a useful natural opportunity for examining the contributions of different compartments. Vasectomy appears to produce only modest changes in seminal microbial community structure, with substantial interindividual variability prevailing. In addition, more than 60% of bacterial communities detected in semen are also detected in the urine, suggesting an important contribution from the urinary tract and other upstream genitourinary compartments [66].

Seminal microbiome profiling requires contamination-controlled approaches because microbial DNA biomass is typically low. Choice of the 16S ribosomal RNA (16S rRNA) region can alter taxonomic profiles, and low biomass makes semen especially vulnerable to reagent and laboratory contamination [67,68,69]. In testicular sperm samples, a large fraction of reads may represent contaminants before decontamination [70]. Cross-site comparisons likewise vary across studies, reinforcing the need for rigorous controls in seminal microbiome work [64,66]. The depth of sequencing and the selected 16S rRNA variable region affect detection and taxonomic classification; 16S amplicon sequencing profiles targeted marker regions, whereas shotgun metagenomics can provide broader genomic information when sufficient microbial sequence coverage is achieved [67,68,69]. Antibiotic exposure, abstinence interval, genital-tract inflammation, sampling materials, and storage conditions may contribute to variation among semen specimens. Extraction blanks, collection controls, absolute bacterial quantification, and culture- or viability-informed analyses help characterize the origin and biological state of detected signals [71].

## 3. Metabolic Disease-Associated Remodeling of the Seminal Microenvironment

### 3.1. Cellular Constituents (Leukocytes and Immature Germ Cells) and Inflammatory Mediators

**Figure 3:**
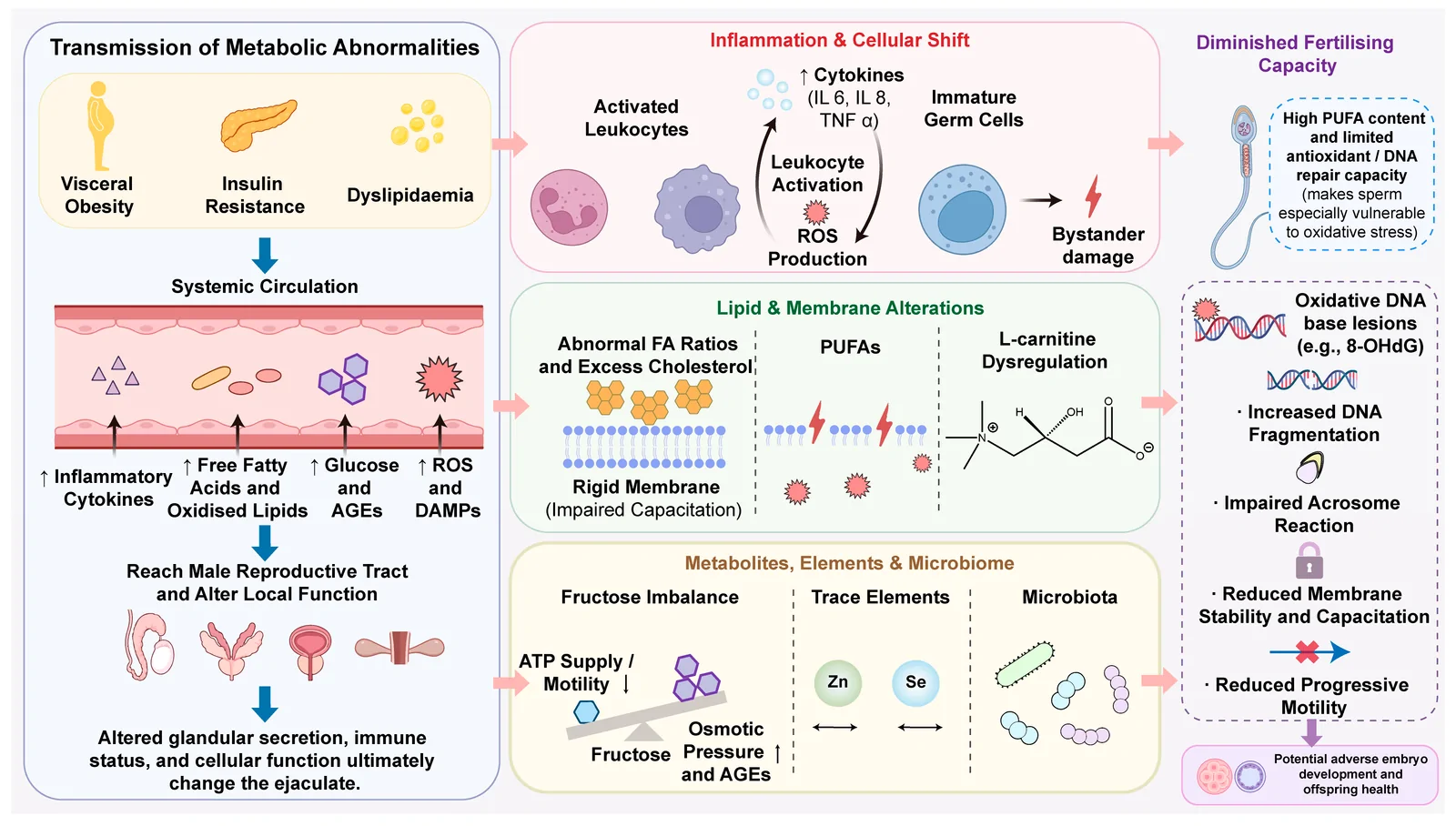
Proposed multidimensional remodeling of the seminal microenvironment under metabolic stress. Visceral obesity, insulin resistance, and dyslipidemia may alter circulating inflammatory cytokines, free fatty acids, oxidized lipids, excess glucose, advanced glycation end products, reactive oxygen species (ROS), and damage-associated molecular patterns. These factors may reach the male reproductive tract and modify glandular secretion, local immune activity, and cellular function. Within semen, metabolic stress may be associated with leukocyte activation, inflammatory cytokines, ROS production, and immature germ cells. In parallel, altered fatty-acid ratios, excess cholesterol, polyunsaturated fatty acid (PUFA) peroxidation, and L-carnitine dysregulation may affect sperm membrane function. Metabolic and microbial alterations may also involve fructose imbalance, changes in trace elements, and seminal microbial profiles. The high PUFA content and limited antioxidant and DNA-repair capacity of sperm increase their susceptibility to oxidative injury. Potential downstream consequences include oxidative DNA base lesions, such as 8-hydroxy-2′-deoxyguanosine (8-OHdG), increased DNA fragmentation, impaired acrosome reaction, reduced membrane stability and capacitation, and lower progressive motility. Possible adverse effects on embryo development and offspring health are also illustrated. Pink horizontal arrows illustrate the proposed progression from systemic metabolic disturbances through alterations in the seminal microenvironment to sperm-related outcomes. Blue downward arrows trace the systemic-to-local route, black arrows indicate proposed local processes or interactions, and the purple downward arrow denotes possible consequences for embryo and offspring health. Upward and downward symbols indicate increases and decreases, respectively.

Seminal leukocytes, cytokines and other humoral constituents are measurable indicators of reproductive-tract immune activity. Metabolic stress may alter these components together with lipids, metabolites and microbial signatures (Figure 3). Different microbial or metabolic signals can engage overlapping innate recognition pathways, although many detailed pathway data come from cultured cells or animal tissue [72,73,74]. A cross-sectional study of 382 middle-aged men reported a modest positive association between BMI and seminal white blood cell count (Spearman’s ρ = 0.112, *p* = 0.030) [75]. Because this was primarily a prostate-screening population, age and urogenital inflammation are relevant covariates when interpreting the association.

Prematurely shed spermatids and spermatocytes are an additional potential source of ROS detected in semen. These immature cells arise during testicular spermatogenesis and retain cytoplasmic components that support reduced nicotinamide adenine dinucleotide phosphate (NADPH)-dependent ROS production [76,77,78]. Their shedding in metabolic disease requires further investigation in metabolically stratified human cohorts.

Inflammatory mediators such as interleukin-6 (IL-6), IL-8 and tumor necrosis factor-α (TNF-α) have been detected at higher concentrations in seminal plasma in some cohorts of infertile men [79]. An experimental study in obese mice addressed a different anatomical compartment: IL-6-related suppressor of cytokine signaling 3 (SOCS3)/signal transducer and activator of transcription 3 (STAT3) changes in spermatogonia were accompanied by altered *Zfp637* and *Sox2* expression and impaired differentiation [80]. This testicular-cell result supports a local inflammatory effect on germ-cell development, while the human data directly describe cytokines sampled from ejaculated seminal plasma.

Metabolic syndrome studies show heterogeneous seminal cytokine profiles: one human case–control cohort reported higher inflammatory mediators, whereas another observed no discriminating seminal cytokine network [81,82,83]. Mouse models of high-fat diet (HFD) exposure identified inflammatory signaling in prostate and seminal-vesicle tissues, including nuclear factor-κB (NF-κB)- and STAT3-related changes [84,85].

### 3.2. Lipids, Metabolites and Glandular Secretions

Sperm function depends on a regulated balance of cholesterol, phospholipids, and polyunsaturated fatty acids (PUFAs); metabolic disruption of these lipids may alter membrane biophysics and increase susceptibility to peroxidation [86,87,88]. The relationship between metabolic state and seminal lipid levels has been analyzed from different perspectives in clinical cohorts. A study examining the relationship between BMI and sperm parameters revealed reductions in total sperm count, sperm concentration, and semen volume in men with abnormal weight. Metabolomics analysis of seminal plasma showed higher spermidine and spermine concentrations in obese men with sperm defects [89]. L-carnitine, which is essential for fatty acid metabolism, was correlated positively with semen quality. Conversely, higher BMI was associated with poorer semen quality and alterations in sperm fatty acid composition [90]. Through subsequent quantitative analyses, palmitic acid (median 24.6 wt%) and docosahexaenoic acid (DHA; median 23.4 wt%) emerged as the two principal sperm fatty acids. After adjustment for covariates, free L-carnitine levels in seminal plasma correlated positively with sperm palmitic acid (*p* = 0.014), DHA (*p* = 0.007), and total n-3 PUFA (*p* = 0.008) levels. Meanwhile, they were correlated negatively with lignoceric acid (*p* = 0.001) and total n-6 PUFA (*p* = 0.012) levels [90]. These findings indicated that seminal metabolites are systematically associated with sperm membrane fatty acids. They provide a measurable association between two different compartments, but not direct evidence of transfer from seminal plasma to the sperm membrane.

Evidence from dietary and pre-clinical studies broadly supports a deleterious role for saturated fatty acids and a protective role for n-3 PUFAs. However, no corresponding shift in the seminal plasma lipid profile has been directly documented in men with obesity. Altered stearic acid and PUFA levels have been reported in both spermatozoa and seminal plasma from infertile patients [91]. In animal models, DHA and n-3 PUFA concentrations correlate positively with sperm viability, survival, normal morphology, and preservation of the plasma membrane. In contrast, the proportion of saturated fatty acids and the n-6/n-3 PUFA ratio correlate negatively with these parameters [92]. Although obesity-related remodeling of the seminal lipidome was not assessed directly, the findings of these studies are consistent with previous evidence. Higher saturated-fat intake has been linked to lower sperm concentration, whereas higher dietary n-3 PUFA intake shows associations with a modestly greater proportion of morphologically normal sperm [93]. However, these findings were obtained from cross-sectional studies that did not measure fatty acids in seminal plasma or sperm membranes. Hence, they cannot directly prove that dietary fats affect semen quality via remodeling of the seminal lipid environment.

Seminal lipid alterations are likely to be caused by both circulatory transfer and altered local metabolism. Sperm fatty acid composition can, for example, be predicted from peripheral erythrocyte lipids. In men with idiopathic infertility, erythrocyte eicosapentaenoic acid (EPA), DHA, the omega-6/omega-3 ratio, and arachidonic acid levels can predict sperm EPA levels, reflecting how systemic lipid status affects the composition of reproductive cells [94]. Conversely, studies show that obesity may directly disrupt sperm lipid handling. In morbidly obese patients, altered expression of lipid receptors and transporters, together with the absence of the 130-kDa hormone-sensitive lipase isoform, may impair the hydrolysis of internalized cholesterol esters. This could promote their accumulation within the sperm cytoplasm and contribute to lipotoxicity and infertility [95]. The lipid-derived mediators found within seminal plasma may also have inherent functional consequences. ROS can induce the release of PUFAs from sperm membranes into seminal plasma, where they may be transformed into prostaglandins and/or isoprostanes. This process may be counteracted by seminal antioxidant enzymes. In infertile men, the seminal prostaglandin E2/6-keto-prostaglandin F_1_α ratio shows a weak but significant inverse correlation with progressive motility (r = −0.22, *p* < 0.001). This suggests that an altered prostaglandin balance may contribute to impaired sperm motility [96]. Metabolomic studies have further indicated that reduced levels of citrate and other key metabolites are associated with impaired sperm function. In contrast, the elevated concentrations of metabolites such as carnitine may reflect disrupted fatty acid metabolism [97].

Environmental pollution offers a complementary, but etiologically distinct, example of seminal biochemical perturbation. In an EcoFoodFertility biomonitoring study of 110 healthy men from Italian areas with different environmental pressures, seminal trace-element profiles differed, accompanied by lower total antioxidant capacity and glutathione and greater sperm DNA fragmentation in the higher-exposure area [98]. More recently, a comparison of 83 men from a per- and polyfluoroalkyl substance (PFAS)-affected area and 50 men from a reference area found higher seminal lipoperoxides and lower antioxidant activity in the exposed group, despite conventional semen parameters within reference limits [99]. These observational findings link environmental context with measurable seminal redox changes.

In a 24-h experimental exposure study, Mediterranean mussels (*Mytilus galloprovincialis*) were exposed to copper, nickel, cadmium, or a metal mixture. Gas chromatography–mass spectrometry identified 237 metabolites in spermatozoa [100]. Malic acid abundance increased after copper, nickel, and combined-metal exposure and decreased after cadmium exposure. Glucose and serine abundance increased after copper, nickel, and combined-metal exposure. This nonmammalian experimental model demonstrates metal-dependent differences in sperm central-carbon metabolites.

Seminal-vesicle-derived fructose supports sperm energy metabolism after ejaculation and marks seminal-vesicle function [34,38]. Its concentration may respond to substrate availability, androgen signalling and glandular dysfunction. Fructose-associated polyol and glycation pathways can produce advanced glycation end products and influence cellular oxidative stress [101,102]. In a human observational study involving 60 non-obese, non-diabetic, and non-smoking men, higher sperm-surface expression of the receptor for advanced glycation end products (RAGE) was associated with reduced motility, lower mitochondrial membrane potential, and greater DNA fragmentation [103]. The impact of metabolic disease on seminal fructose itself requires direct measurement. Seminal zinc is commonly measured as a prostatic secretory and reproductive biomarker. A meta-analysis of 20 studies involving 2600 infertile men and 867 controls reported lower seminal zinc in infertile men (SMD = −0.64, 95% CI −1.01 to −0.28) [104]. Other clinical and experimental reports link zinc-related interventions or measurements to sperm motility, morphology or chromatin indices [105,106,107]. The seminal metallome also contains selenium, magnesium, calcium, copper and manganese, which have been studied in relation to sperm function [108]. Selenium and selenoprotein *p* have been associated with antioxidant activity or semen quality [109,110]. Most of these studies group men by fertility status; direct metabolic phenotype-specific measurements remain limited.

### 3.3. Epididymal Extracellular Vesicles

Epididymal EVs transfer molecular cargo to sperm during post-testicular maturation. In HFD-fed mice, epididymal oxidative and endoplasmic-reticulum stress was accompanied by 202 differentially abundant proteins in epididymal EVs, elevated CD63 expression, increased sperm ROS, and reduced sperm motility [111]. Palmitate exposure also promoted exosome secretion by cultured epididymal epithelial cells [111]. In a functional mouse study, epididymal epithelial-specific *Dgcr8* deletion reduced 27 sperm microRNAs and altered early-embryo gene expression, which was rescued by microinjection of epididymal microRNAs [112]. In a separate corticosterone-stress model, incubating unexposed sperm with EVs from treated epididymal epithelial cells altered offspring neurodevelopment and stress responses after intracytoplasmic sperm injection (ICSI) [113]. Together, these findings link metabolic stress with epididymal EV remodeling and demonstrate that epididymal molecular cargo can influence post-fertilization outcomes.

### 3.4. Seminal Microbiome Alterations

Evidence for metabolic disease-associated changes in the seminal microbiota remains limited. In mice, HFD consumption has been shown to alter the microbial composition of both the feces and seminal fluid. This indicates that diet-responsive microbial changes can occur concurrently in the intestinal and reproductive compartments [114]. Small human observational cohorts have also linked seminal microbial profiles with BMI, sperm DNA fragmentation and antioxidant capacity [115,116] (Figure 3).

Gut microbiome studies have provided complementary yet mechanistically distinct evidence. HFD-induced gut dysbiosis can impair spermatogenesis and sperm motility via systemic inflammatory, endocrine, and metabolic pathways [117]. Partial taxonomic overlap among gut, urine, and semen samples has also been detected through cross-site profiling [28,64,66]. These findings reflect the presence of coordinated microbial and host responses across anatomical compartments. However, they do not prove that bacteria directly migrate from the intestine to the seminal tract. Instead, inflammation, circulating microbial products, altered barrier function, and changes in host metabolites may indirectly mediate the influence of gut dysbiosis on the reproductive-tract environment.

In a human observational study of 13 infertile and five fertile men with obesity, 16S sequencing identified associations of selected seminal genera with BMI, sperm DNA fragmentation and seminal total antioxidant capacity [115]. In a single-center observational study involving 45 men undergoing assisted reproduction, seminal microbial diversity varied with BMI, dietary and smoking-related variables, and *Staphylococcus* spp. was more abundant in the group without pregnancy during follow-up [116]. An observational metagenomic study of infertile couples found niche-specific microbial profiles across semen, penile, vaginal and follicular-fluid specimens [118]. These observations identify metabolic state, reproductive outcomes and genital-site exposures as overlapping correlates of seminal microbial profiles. Genital-tract inflammation, medication or antibiotic use, abstinence interval and sample collection can also influence the local environment or detected microbial signals [61,71]. One testable hypothesis is that changes in semen quality or genital-tract inflammation precede some of the microbial DNA profiles measured in the ejaculate. Longitudinal within-person sampling, paired-partner sampling and absolute bacterial quantification can determine the temporal relationships among metabolic state, microbial profiles and sperm function. The evidence for metabolic remodeling across the major components of the seminal microenvironment is summarized in Table 1.

## 4. Sperm Dysfunction in the Altered Seminal Microenvironment

### 4.1. Oxidative Stress and Inflammatory Amplification

**Figure 4:**
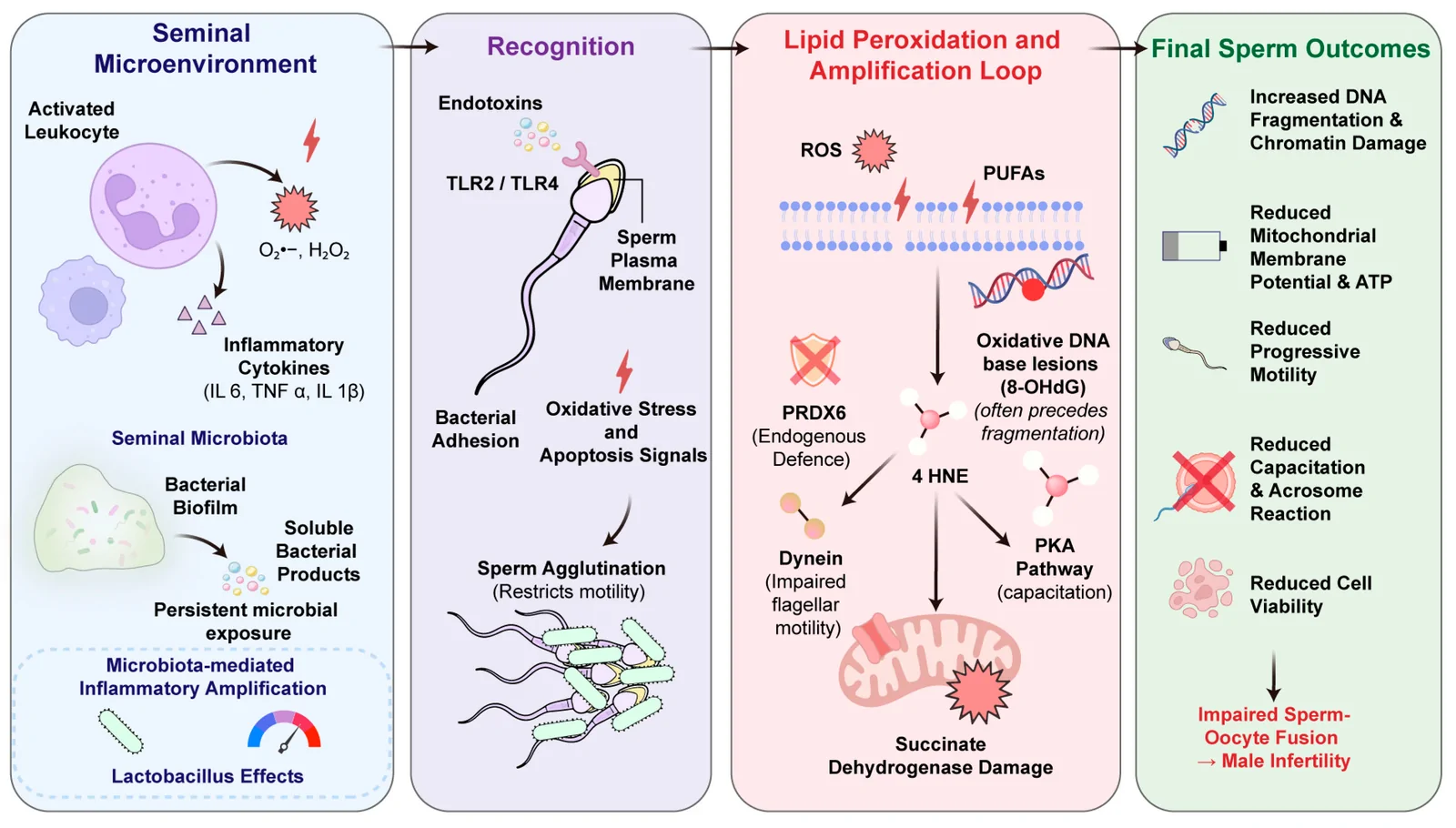
Mechanistic pathways by which oxidative, inflammatory, and microbial disturbances in the seminal microenvironment may impair sperm function. Activated seminal leukocytes release reactive oxygen species (ROS) and inflammatory cytokines, whereas seminal bacteria may act through adhesion, agglutination, biofilm persistence, soluble products, and inflammatory amplification. Microbial products can activate sperm Toll-like receptor 2 and Toll-like receptor 4, promoting oxidative and apoptotic signaling, while bacterial adhesion and agglutination can restrict progressive motility. These insults converge on membrane lipid peroxidation, in which ROS attack polyunsaturated fatty acids (PUFAs) and generate electrophilic aldehydes such as 4-hydroxynonenal (4-HNE). Oxidative DNA base lesions, including 8-hydroxy-2′-deoxyguanosine (8-OHdG), may develop before DNA strand fragmentation. Peroxiredoxin 6 (PRDX6) provides endogenous protection against lipid peroxidation, whereas excessive 4-HNE can modify proteins involved in flagellar movement and capacitation and impair succinate dehydrogenase, increasing mitochondrial electron leakage and secondary ROS generation. This amplification loop links membrane injury to mitochondrial dysfunction. Downstream consequences include increased DNA fragmentation and chromatin damage, reduced mitochondrial membrane potential and ATP production, impaired motility, defective capacitation and acrosome reaction, reduced viability, and compromised sperm–oocyte interaction. *Lactobacillus*-associated effects depend on the bacterial strain and exposure conditions. Black arrows indicate the proposed direction of molecular interactions, damage propagation, and downstream functional consequences.

Once they are activated within the seminal compartment, polymorphonuclear leukocytes become a major source of oxidative injury. At equivalent cell numbers, activated leukocytes can generate approximately 1000-fold more ROS than spermatozoa. The resulting extracellular oxidants can contact sperm already present in ejaculated semen and induce membrane lipid peroxidation, protein oxidation, and DNA damage [122,123] (Figure 4). Importantly, oxidative injury may even occur below the conventional threshold associated with leukocytospermia. For instance, men with low-level leukocytospermia have been found to show higher seminal ROS levels and greater DNA damage in sperm even in the presence of other normal semen parameters [124]. Evidence suggests that the adverse effects of leukocyte accumulation may become detectable at concentrations above approximately 0.2 × 10^6^ leukocytes/mL [125,126,127,128]. A human cross-sectional study also linked greater adiposity with an increased seminal leukocyte burden [75]. Rising seminal leukocyte burden has been associated with poorer semen quality, chromatin disruption, and DNA fragmentation. ROS appears central to this relationship, and leukocyte-derived oxidants can damage sperm lipids, proteins, and DNA [46,129].

A meta-analysis comparing infertile men with fertile controls reported higher seminal IL-6 (SMD = 0.39, 95% CI 0.14–0.64), TNF-α (SMD = 0.13, 95% CI 0.00–0.25) and IL-8 (SMD = 0.24, 95% CI 0.06–0.43), with heterogeneity differing among markers [130]. Cytokine-related oxidative, apoptotic and mitochondrial responses have been described in reproductive-tract models [131,132,133,134,135]. In an ex vivo exposure study of ejaculated human spermatozoa, TNF-α altered sperm motility and apoptosis-related measurements after sperm formation [136]. In men with intractable spermatocystitis, inflammatory and ROS-related findings were accompanied by lower nuclear factor erythroid 2-related factor 2 (Nrf2) protein abundance and reduced levels of its phosphorylated form [137]. These studies describe separate seminal, mature-sperm and accessory-gland settings within the shared inflammatory–redox framework.

### 4.2. Lipid Peroxidation, Membrane Injury and Mitochondrial Dysfunction

Sperm membranes are rich in PUFAs, including DHA, and have limited repair capacity. Their multiple double bonds provide substrates for free-radical chain propagation during oxidative exposure [86,87,88]. Thus, the metabolic remodeling of membrane lipids can alter membrane biophysics while simultaneously increasing vulnerability to lipid peroxidation. During the epididymal transit, sperm maturation occurs. This process involves the extensive remodeling of membrane phospholipids, including an increase in DHA and the PUFA/saturated fatty acid ratio in humans. In mice, targeted disruption of *Pla2g3*, which encodes group III secreted phospholipase A_2_ in the proximal epididymal epithelium, compromises epididymal sperm membrane lipid remodeling, causes sperm structural and motility defects, and markedly impairs fertilization capacity [90,138]. This model demonstrated that changes in the lipid environment induced by the reproductive-tract epithelium can also alter sperm membrane fatty acid composition and compromise fertilizing capacity.

Metabolic disease appears to disrupt sperm lipid handling directly. In morbidly obese patients, altered expression of lipid receptors and transporters, together with loss of the 130-kDa hormone-sensitive lipase isoform, can impair the hydrolysis of internalized cholesterol esters and promote their accumulation within the sperm cytoplasm. This process may contribute to lipotoxicity and infertility [95]. Animal models provide further causal evidence at the membrane level. In New Zealand white rabbits, increasing dietary cholesterol by 0.05% (*w*/*w*) significantly increased serum and sperm membrane cholesterol and altered membrane-dependent sperm functions, including osmotic resistance, the acrosome reaction and capacitation [139]. Meanwhile, diet-induced hypercholesterolemia also impaired semen quality, motility, capacitation, and the acrosome reaction, probably through an increase in cellular cholesterol levels and consequent disruption of membrane-dependent processes [140]. These findings reflect a mechanistic pathway linking dietary lipid intake to circulating lipids, sperm membrane cholesterol, and impaired capacitation within a single experimental system. In humans, normozoospermic men belonging to infertile couples may also exhibit abnormal seminal and circulating fatty acid profiles, with increased lipid peroxidation, despite conventional semen parameters remaining within reference ranges. Lower concentrations of very-long-chain PUFAs in sperm cells have also been linked to reduced sperm count and motility [141].

An altered lipid environment may affect sperm through membrane cholesterol exchange and susceptibility to peroxidation. Experiments in bovine epididymal spermatozoa exposed to seminal-plasma proteins demonstrated cholesterol and phospholipid efflux after sperm formation [142,143]. Higher BMI was associated with lower sperm DHA in a human cohort, and other human studies examined lipid-related membrane anisotropy [120,144]. However, these measurements came from distinct systems.

Among the oxidative mediators within the seminal microenvironment, 4-hydroxynonenal (4-HNE) has particular analytical and mechanistic relevance. 4-HNE is a relatively stable product of lipid peroxidation, can be measured in seminal plasma, and forms protein adducts through defined chemical reactions. It can therefore serve as a useful molecular link between oxidative injury and sperm dysfunction. Excess ROS accumulation promotes the peroxidation of membrane PUFAs through free-radical chain reactions, generating bioactive aldehydes such as 4-HNE [145]. Other aldehydic products of lipid peroxidation include acrolein and malondialdehyde; in human spermatozoa, acrolein and 4-HNE show marked bioactivity by impairing motility and increasing ROS generation, lipid peroxidation, oxidative DNA damage, and caspase activation, whereas malondialdehyde is better interpreted as a correlated marker of oxidative stress [146,147].

In ex vivo experiments involving mature human spermatozoa, electrophilic aldehydes such as 4-HNE and acrolein stimulated mitochondrial superoxide generation in a concentration- and time-dependent manner. 4-HNE targeted succinate dehydrogenase activity, increasing electron leakage and producing loss of mitochondrial membrane potential and apoptosis-related events [148]. This study describes a direct post-testicular exposure that links lipid-peroxidation products with mitochondrial dysfunction. The resulting secondary ROS may amplify aldehyde formation and oxidative damage to additional sperm components. Second-order effects have also been detected. These include the adduction of the motility apparatus and disruption of capacitation signaling. 4-HNE can impair sperm motility by forming adducts with proteins required for flagellar movement, including dynein [149]. Furthermore, it can promote superoxide-dependent peroxynitrite formation and protein tyrosine nitration. Accordingly, it can disrupt the signaling required for motility and capacitation. Endogenous antioxidant systems can combat lipid-peroxidation injury. The mouse *Prdx6* gene encodes peroxiredoxin 6 (PRDX6), a bifunctional protein with peroxidase and calcium-independent phospholipase A_2_ activities. These activities limit lipid-peroxidation-mediated damage. Inhibition of PRDX6 activity or mutation of the encoding gene increases sperm lipid-peroxidation products, including 4-HNE. Thus, it is associated with mitochondrial dysfunction, impaired motility and capacitation, DNA oxidation and subfertility [150,151]. These findings demonstrate the functional importance of limiting lipid peroxidation.

In a human observational study, infertile men had higher seminal 4-HNE, DNA fragmentation and caspase-3 activity than normozoospermic men (*p* < 0.001) [152]. Seminal ROS correlated with 4-HNE (r = 0.46), caspase-3 (r = 0.53) and sperm DNA fragmentation (r = 0.48; all *p* < 0.001). Human sperm experiments further identified 4-HNE adducts on proteins involved in flagellar movement and capacitation [148,149,153,154]. These clinical correlations and laboratory mechanisms support investigation of 4-HNE as an oxidative injury mediator. Direct metabolic disease-specific measurements of seminal 4-HNE and reproducible exposure–response relationships with human sperm functional endpoints are still needed [155,156].

### 4.3. Sperm DNA Damage and Post-Testicular Epigenetic Changes

Sperm DNA fragmentation can develop during spermatogenesis, epididymal transit or later extracellular exposure. Mature human sperm retain recognition of oxidized guanine by 8-oxoguanine DNA glycosylase 1 (OGG1) but have restricted downstream base-excision repair; 8-hydroxy-2′-deoxyguanosine (8-OHdG) and abasic intermediates may therefore precede DNA strand breaks [157,158]. These stages are relevant when attributing DNA damage measured in ejaculated sperm to a specific exposure window.

EcoFoodFertility studies have also assessed sperm nuclear proteins and small RNAs in environmentally exposed populations. In young men from areas with contrasting pollution burdens, sperm nuclear basic proteins showed differences in protamine–histone composition and DNA-binding properties; proteins isolated from selected samples were associated with oxidative DNA damage in experimental assays [159]. In a further observational study involving 33 men, sperm nuclear-protein profiles and several small noncoding RNA species differed between residential groups [160]. These findings describe exposure-related chromatin and RNA measurements in human sperm.

Human observational research has reported differences in ejaculated sperm DNA methylation after obesity or bariatric surgery [161,162]. Experimental epididymal systems have documented RNA-containing vesicles and the acquisition of selected sperm RNAs [112,163,164,165]. In obese mice, epididymal EV cargo changed [111], while short paternal HFD exposure altered mitochondrial small RNAs in cauda-epididymal sperm and was linked to offspring metabolic phenotypes [166].

## 5. Microbial Injury and Downstream Consequences Within the Seminal Microenvironment

The strongest evidence linking the seminal microbiota to sperm dysfunction has been obtained for general microbial processes. However, evidence for genus-specific mechanisms is much more limited. Thus, this section first discusses functional evidence, followed by the taxa associated with adverse or favorable sperm outcomes.

### 5.1. Experimentally Supported Mechanisms of Microbial Sperm Injury

Direct adhesion and agglutination: Evidence for this process comes primarily from bacteriospermia studies and co-incubation models [167,168]. Studies show that bacterial cells can bind to the surface of sperm cells and form sperm–bacterium aggregates. This physically restricts the progressive movement of sperm and increases their exposure to microbial products.

Soluble bacterial products and mitochondrial-membrane injury: Sperm injury can occur even in the absence of direct contact with bacterial cells. For instance, in an ex vivo study involving ejaculated human spermatozoa, soluble factors released by *Escherichia coli* can reduce mitochondrial membrane potential, promote phosphatidylserine externalization, and decrease sperm viability and motility [169].

Recognition of bacterial products by sperm Toll-like receptors: Human spermatozoa express both Toll-like receptor 2 and Toll-like receptor 4. Exposure to bacterial endotoxins can activate these receptors and increase markers of oxidative stress and apoptosis. This alters sperm motility and the acrosome reaction [170,171].

Leukocyte-mediated bystander injury: The presence of microbes in the male reproductive tract can trigger the recruitment and activation of seminal leukocytes. The resulting release of ROS, cytokines, and proteases causes membrane lipid peroxidation, mitochondrial dysfunction, and DNA damage in neighboring spermatozoa [123,129,168,172]. This pathway overlaps with the inflammatory mechanisms described above.

Biofilm persistence: Biofilms can limit drug penetration and decrease bacterial metabolic activity. Hence, they can prolong bacterial residence and reduce antimicrobial responsiveness in bacterial cells. The presence of biofilm-forming isolates in semen has been linked to altered cytokine regulation. Experimental models of *Enterococcus faecalis* bacteriospermia have demonstrated the persistent exposure of spermatozoa to biofilms [172,173]. Biofilm formation therefore provides a more direct explanation for microbial persistence than for direct molecular injury to spermatozoa.

Together, these mechanisms converge on the sperm membrane, mitochondria, and surrounding inflammatory milieu. Capacitation requires cholesterol efflux, preserved membrane fluidity, intracellular alkalinization, and Ca^2+^-dependent signaling. Microbial adhesion, oxidative membrane injury, and inflammatory exposure can therefore impair sperm hyperactivation and the acrosome reaction. However, a complete pathway linking any specific bacterial taxon to impaired sperm capacitation is yet to be delineated within a single experimental system [168,169,170,171].

### 5.2. Taxa Associated with Adverse Semen and Sperm Endpoints

Genus-level associations between the seminal microbiota and semen quality have been reported across cohort and meta-analytic studies. *Prevotella*, *Enterococcus*, *Anaerococcus*, *Ureaplasma*, *Mycoplasma*, *Pseudomonas*, and several anaerobic taxa have been associated with one or more adverse sperm endpoints, including lower sperm concentration, impaired motility, abnormal morphology, increased oxidative stress, and sperm DNA fragmentation [62,172,174,175].

*Prevotella*: *Prevotella*-enriched profiles have been repeatedly detected in semen samples. Higher *Prevotella* abundance has been associated with lower sperm motility, reduced sperm concentration, oligospermia, impaired DNA integrity, and altered redox homeostasis [64,174,176,177].

*Enterococcus*: Studies of human semen samples have associated higher *Enterococcus* abundance with lower sperm concentration, reduced progressive motility, altered seminal composition, and increased sperm DNA damage [174,175,178,179].

*Anaerococcus*: *Anaerococcus* is repeatedly detected in semen and has been associated with altered sperm quality. In an early cohort study, *Anaerococcus* was the only genus whose association with abnormal semen parameters remained statistically significant after Bonferroni correction. It was also identified as a biomarker distinguishing dysspermatism from control semen samples [62,115,175,180].

Other taxa: *Finegoldia*, *Campylobacter*, *Peptoniphilus*, *Gardnerella*, and some *Corynebacterium* species have been associated with reduced sperm motility, abnormal sperm morphology, increased oxidative stress, or sperm DNA fragmentation [167,174,181].

### 5.3. Lactobacillus-Associated States: Potential Protection and Context Dependences

*Lactobacillus*-enriched seminal profiles have been associated with normal sperm morphology, lower leukocyte counts, reduced oxidative DNA damage, and more favorable semen-quality clusters [174,176,177,182]. In contrast, *Lactobacillus*-containing signatures have also been reported in azoospermia and other forms of dysspermatism [180,183]. *Lactobacillus iners* was enriched in semen samples with abnormal motility and was identified as a discriminator of abnormal combined semen parameters [184].

In vitro studies have shown that soluble products from selected strains of *Lactobacillus brevis*, *Lactobacillus salivarius*, and *Lactiplantibacillus plantarum* (*formerly Lactobacillus plantarum*) reduce experimentally induced membrane lipid peroxidation in sperm cells and preserve sperm motility [185]. Postbiotics derived from *Lactobacillus rhamnosus* strain PB01 showed no adverse effects on sperm quality at low concentrations, whereas higher concentrations reduced progressive motility and increased DNA fragmentation [186].

Thus, *Lactobacillus*-associated states may correlate with favorable sperm endpoints, and selected strains can be protective in vitro, but the genus should not be considered uniformly beneficial.

These oxidative, inflammatory, lipid-peroxidation, and microbial pathways converge on overlapping sperm targets and functional outcomes (Table 2; Figure 4).

## 6. Therapeutic Strategies Targeting the Seminal Microenvironment

**Figure 5:**
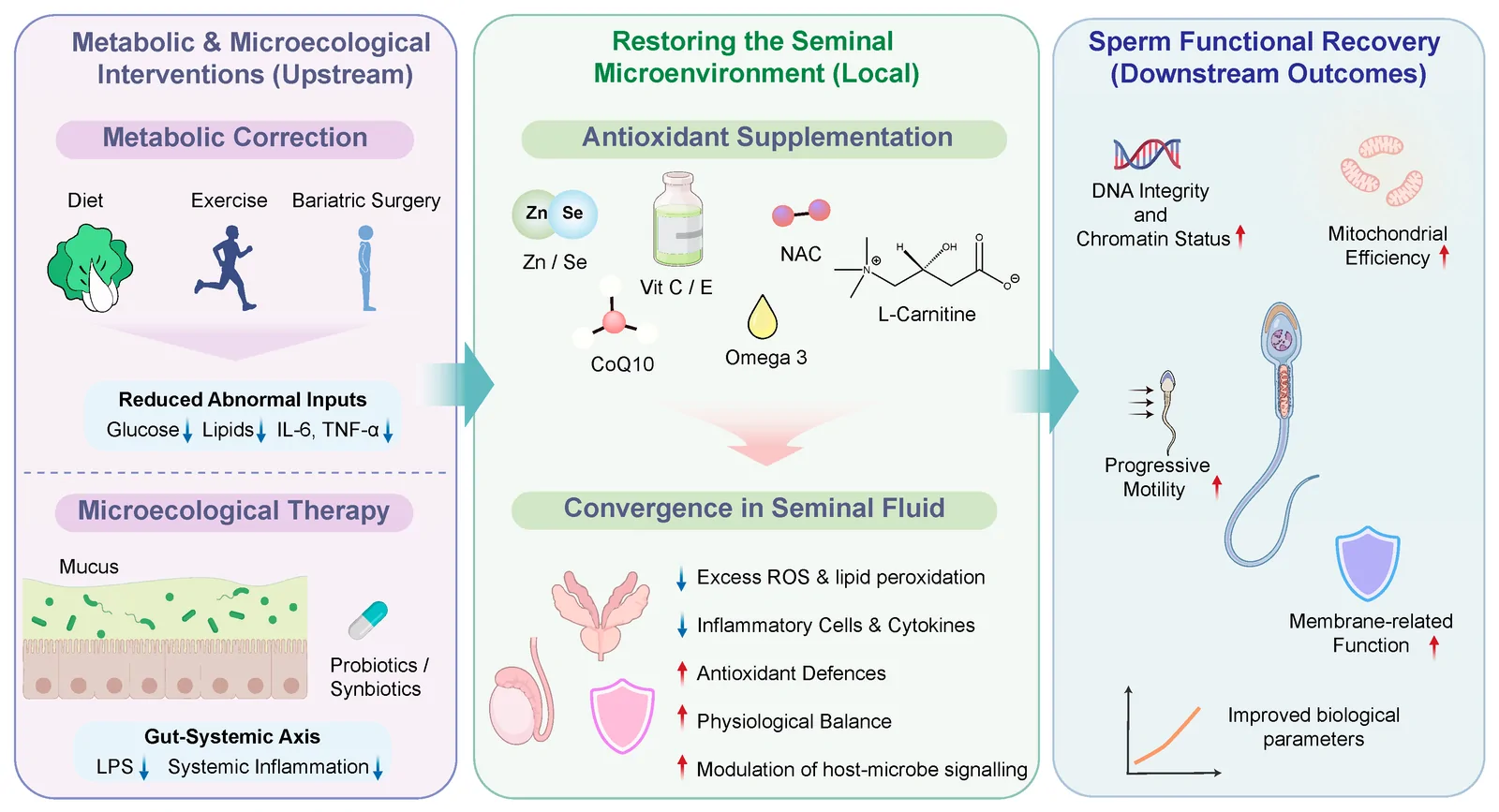
Therapeutic framework for targeting the seminal microenvironment and promoting sperm functional recovery. Upstream metabolic correction includes dietary intervention, structured exercise, and bariatric surgery, which may reduce excess glucose and lipids and lower systemic inflammatory mediators. Microbiome-directed approaches, including probiotics and synbiotics, may act mainly through the gut–systemic axis by improving barrier and metabolic homeostasis and reducing lipopolysaccharide exposure and systemic inflammation; direct remodeling of the human seminal microbiota remains less well established. At the local level, antioxidant supplementation may reinforce seminal redox buffering through zinc and selenium, vitamins C and E, N-acetylcysteine, coenzyme Q10, omega-3 fatty acids, and L-carnitine. These interventions may converge within seminal fluid by reducing excessive reactive oxygen species and lipid peroxidation, limiting inflammatory cells and cytokines, restoring antioxidant defenses and physiological balance, and modulating host–microbe signaling. Potential downstream benefits include improved sperm DNA integrity, mitochondrial efficiency, ATP production, progressive motility, and membrane-related functions. The framework presents the seminal microenvironment as a potentially modifiable preconception interface, while recognizing that direct evidence for improved fertility outcomes remains limited. Large horizontal teal arrows represent the proposed progression from upstream interventions through restoration of the seminal microenvironment to downstream sperm functional outcomes. The central downward arrow indicates convergence on seminal fluid, while blue downward and red upward arrows denote decreases and increases in the indicated components, respectively.

The preceding sections discuss how the seminal microenvironment can act as a dynamic post-testicular interface where metabolic substrates, accessory-gland secretions, redox balance, immune activity, and microbial signals converge on sperm function. This framework creates three complementary therapeutic opportunities (Figure 5). Metabolic correction can reduce the systemic inputs that drive remodeling of the seminal compartment. Meanwhile, antioxidant and nutritional interventions may restore local redox defense and membrane protection. Finally, microbiome-targeted approaches may modify microbial, immune, and metabolic signaling.

### 6.1. Metabolic Correction: Eliminating the Upstream Drivers of Seminal Microenvironmental Remodeling

Metabolic correction addresses the most upstream drivers of seminal remodeling. By reducing excess glucose, lipids, insulin, adipokines, and inflammatory mediators reaching the epididymis and accessory glands, it may permit recovery of glandular secretion, redox balance, and post-testicular sperm maturation.

Dietary and weight-loss interventions provide human evidence for changes in sperm function after systemic metabolic improvement. In an eight-week low-calorie intervention in men with obesity, sperm concentration and total count increased, with benefits maintained in those sustaining weight loss [189]. A 12-week lifestyle program involving 121 men linked an 8.2% BMI reduction to a 13.4% reduction in sperm DNA fragmentation [190]. In 43 men with severe obesity, an approximately 15% BMI reduction was accompanied by improvements in semen volume, total count and morphology [191]. The EcoFoodFertility FASt randomized trial enrolled 344 young men living in polluted Italian areas to assess a four-month Mediterranean diet and physical-activity program; 263 completed follow-up. Selected motility measures and seminal antioxidant capacity were more favorable after intervention [192].

Exercise may act through changes in systemic metabolism, inflammatory burden and reproductive-tract antioxidant activity. A network meta-analysis of randomized studies reported improvements in selected sperm-quality measures with structured exercise [193]. A 24-week randomized treadmill trial in infertile men measured a decline in seminal IL-6, TNF-α, ROS and malondialdehyde alongside improved antioxidant measures, semen parameters and reduced sperm DNA fragmentation [194]. This trial directly linked a behavioral intervention to defined seminal biochemical measurements and sperm-related endpoints, although reproductive outcomes beyond these measurements were not established.

Bariatric surgery offers longitudinal evidence on metabolic change and semen-related outcomes in severe obesity. A prospective double-armed study reported lower seminal IL-8 after surgery, with increases in semen volume and improved sperm vitality and motility and reduced sperm DNA fragmentation [195]. A separate 12-month prospective investigation found reduced seminal lipid peroxidation accompanied by changes in sperm progressive motility, morphology, mitochondrial activity and DNA integrity [156]. In a sleeve-gastrectomy cohort, improved BMI, insulin resistance and lipid metabolism preceded reproductive-function changes detected over approximately 6–12 months [196]. These findings characterize the timing and compartments measured during major metabolic correction.

### 6.2. Antioxidant Supplementation: Restoring Seminal Redox Defense and Limiting Lipid-Peroxidation Injury

Antioxidant supplementation aims to restore physiological redox balance in seminal plasma and sperm by limiting excess ROS, lipid peroxidation and electrophilic aldehyde exposure.

Antioxidant interventions can be classified according to the redox components they primarily support. Zinc and selenium contribute to superoxide dismutase- and glutathione peroxidase-dependent defense, while vitamins C and E protect the aqueous and lipid phases, respectively. In addition, N-acetylcysteine (NAC) and glutathione-related compounds replenish thiol-reducing capacity. Further, coenzyme Q10 (CoQ10) supports electron transport and may limit mitochondrial electron leakage. Finally, L-carnitine facilitates fatty-acid utilization and ATP production, whereas omega-3 fatty acids influence sperm-membrane phospholipids and modulate inflammatory and oxidative signaling. Previous meta-analyses of randomized trials have linked selenium, zinc, omega-3 fatty acids, CoQ10, and carnitines with improvements in one or more measures of sperm quality [107,197]. In infertile men with asthenoteratozoospermia, three months of oral NAC supplementation (600 mg/day) increased seminal total antioxidant capacity, reduced malondialdehyde, improved sperm count and motility, and reduced abnormal morphology, DNA fragmentation, and protamine deficiency [198]. In a double-blind randomized trial involving men with idiopathic oligoasthenoteratozoospermia, CoQ10 supplementation appeared to increase seminal CoQ10 concentrations and improve antioxidant-enzyme activity while reducing oxidative-stress indices [199]. Finally, a randomized trial of DHA with vitamin E also demonstrated coordinated changes in seminal oxidative stress, sperm phospholipids, and the spermatogram in men with asthenozoospermia [200].

A recent mechanistic investigation of food-derived phenols provides a related, but indirect, model of protection against toxicant-induced membrane injury. Perrone et al. reported that hydroxytyrosol, tyrosol, and homovanillyl alcohol reduced mercury-induced phosphatidylserine externalization in human erythrocytes [201]. Mercury chloride suppressed ATP11C flippase activity and increased PLSCR1 scramblase activity, whereas phenol pretreatment counteracted these changes and restored cellular ATP and glutathione; intracellular calcium remained unchanged under the tested conditions. Molecular docking suggested a possible interaction with ATP11C but did not establish direct binding.

Collectively, these findings support antioxidant supplementation as a potential strategy for restoring seminal redox capacity and limiting oxidative DNA damage, membrane instability, mitochondrial dysfunction, and deterioration in conventional sperm parameters.

### 6.3. Microbiome-Targeted Therapy: Restoring Microbial–Immune–Metabolic Homeostasis

Microbiome-targeted therapy aims to modify microbial and host pathways simultaneously. Current human evidence derives mainly from oral probiotics and synbiotics and is stronger for sperm and redox endpoints than for direct remodeling of the seminal microbiome. These approaches should therefore be considered complementary to metabolic correction and redox support.

Human intervention studies on microbiome targeting have been encouraging so far. A systematic review of randomized clinical studies concluded that probiotic or synbiotic supplementation can improve measures of sperm quality and reduce oxidative or DNA damage in men with infertility [202]. In a triple-blinded randomized trial, 80 days of treatment with the synbiotic FamiLact led to increased sperm concentration and motility, improved morphology, reduced DNA damage, and enhanced chromatin and protamine-related indices [203]. Similar findings have also been obtained with strain-specific treatment. For instance, in one study, oral *Lactobacillus rhamnosus* CECT8361 and *Bifidobacterium longum* CECT7347 improved sperm motility and reduced ROS and DNA fragmentation after three to six weeks in men with asthenozoospermia [204]. Complementing these findings, preclinical studies have hinted at broader, multi-level mechanisms. In HFD-induced murine models of obesity, *Lactiplantibacillus plantarum* 1008 was found to reduce circulating lipopolysaccharide levels and insulin resistance, improve testicular antioxidant-enzyme activity, and attenuate lipid peroxidation. These changes were accompanied by improved sperm quality and spermatogenesis [205]. Of course, this model did not confirm the direct modification of the human seminal microbiota following intervention. It did, however, suggest that restoring gut-barrier integrity and systemic immune–metabolic homeostasis can reduce the metabolic and inflammatory burden within the reproductive tract. Postbiotics offer a more standardizable alternative. *Lactobacillus rhamnosus* PB01-derived products show concentration- and time-dependent effects on human sperm, with low concentrations preserving sperm quality but higher concentrations reducing progressive motility and increasing DNA fragmentation [186].

Human probiotic and synbiotic trials have reported improvements in selected sperm parameters and DNA integrity [202,203,204]. These sperm-related findings support further evaluation of the seminal microenvironment as a potentially modifiable interface for mechanism-directed preconception interventions (Figure 5). The study designs, biochemical measurements, and sperm endpoints reported across these human intervention studies are summarized in Table 3.

## 7. Discussion and Future Perspectives

### 7.1. Metabolic Phenotypes and Candidate Seminal Biomarkers

Human studies report associations between metabolic state and seminal metabolites, lipids, adipokines, microRNAs and glycosylation-related markers [90,119,206,207,208,209]. These molecular measurements cover multiple reproductive compartments and endpoints. Experimental studies of mature ejaculated sperm and epididymal sperm provide post-testicular evidence for lipid exchange, mitochondrial responses and changes in sperm function. Table 4 compares obesity, insulin resistance, type 2 diabetes mellitus, dyslipidemia and metabolic syndrome according to their measured seminal or sperm findings, reproductive endpoints and study designs.

Human studies have linked seminal metabolites, lipids, cytokines, EV-associated RNAs and microbial signatures to sperm quality and reproductive outcomes. An eight-metabolite seminal-plasma panel distinguished teratozoospermic men from normozoospermic controls, with an area under the receiver operating characteristic curve (AUC) of 0.881 [214], while EPA distinguished infertile from proven-fertile men (AUC = 0.709) [86]. In assisted reproduction, seminal interleukin-18 (IL-18) was associated with ICSI failure (AUC = 0.828) [215], and lower transforming growth factor-β (TGF-β) concentrations were observed in the live-birth group [216]. Seminal EV profiling identified 10 circular RNAs and two piRNAs differing between couples with and without a live birth [217]. Prospective metagenomic data linked altered seminal microbial profiles to a lower rate of achieving live birth after in vitro fertilization (IVF) or ICSI, with a hazard ratio (HR) of 0.37 (95% CI 0.19–0.71) [218]. A systematic review of 43 studies identified 32 candidate seminal-plasma biomarkers for IVF/ICSI outcomes, with 23 reported in a single study [219]. The MiOXSYS assay demonstrated analytical reproducibility of seminal oxidation–reduction potential measurements across operators, instruments and testing days [220]. Repeated sampling, standardized assays and externally validated prediction of pregnancy and live birth in metabolically stratified couples are needed to assess the clinical value of these markers for preconception care.

### 7.2. Paternal Metabolic Health and Reproductive Outcomes

Paternal metabolic health has been assessed in human assisted-reproduction cohorts and in experimental models of offspring development. In a retrospective study of 7618 couples undergoing IVF or ICSI, clinical pregnancy rates were 59.2%, 56.6% and 55.5% across normal-weight, overweight and obese paternal BMI groups, respectively; normal-weight men also had higher rates of usable embryos or blastocysts in selected comparisons [221]. A prospective observational time-lapse study of 1210 embryos found that paternal BMI was associated with the timing of pronuclear appearance and selected early divisions, including t2 and t8, after adjustment for maternal factors [222]. In mice, two weeks of paternal HFD exposure altered mitochondrial transfer RNA-derived small RNAs in cauda-epididymal sperm; sperm-borne mitochondrial RNAs were detected in early embryos, and male offspring showed altered glucose regulation [166]. In the human LIFE Child cohort (*n* = 3431), the same study reported associations of paternal BMI at conception with offspring adiposity and metabolic measures [166]. These findings characterize human embryo and pregnancy associations, human parent–offspring metabolic associations, and a diet-responsive sperm RNA pathway with offspring phenotypes in mice. Future integrated studies can assess paternal metabolism, defined seminal and sperm molecular measurements, early embryo responses and offspring outcomes in a single longitudinal framework.

### 7.3. Evidence Boundaries and Unresolved Causal Relationships

Current evidence has five main limitations: temporal ordering, mediation by defined seminal factors, post-testicular molecular attribution, low-biomass microbial measurement, and the clinical effects of antioxidant supplementation.

First, the temporal relationship between metabolic disease and seminal remodeling remains unresolved. Obesity and metabolic syndrome have been associated with alterations in seminal cytokines, adipokines, metabolites, and microRNAs [81,119,206,207], but most evidence is cross-sectional. Few intervention studies have assessed systemic metabolic changes, changes in the seminal microenvironment, and sperm function in parallel, making it difficult to determine whether seminal remodeling precedes sperm recovery. Longitudinal within-person studies are therefore required to establish the direction and sequence of these changes. Associations between metabolic phenotypes, seminal-plasma constituents, and sperm measurements can be influenced by characteristics of the sampled population. Age, recent fever, and varicocele affect sperm-related clinical measurements, including motility and DNA integrity; genital infection and inflammation can modify seminal leukocyte and cytokine profiles [55,57,75]. Smoking, alcohol intake, diet, and physical activity contribute to variability in systemic metabolic and oxidative status [93]. Medication use, particularly antibiotics, can also influence microbial signals in semen, while abstinence duration affects ejaculate sampling measures. Socioeconomic conditions may correlate with lifestyle and environmental exposures. Human observational studies can therefore report these factors and adjust for them when estimating associations between metabolic characteristics and seminal endpoints.

Second, experiments on epididymal or ejaculated sperm have demonstrated responses to selected seminal-plasma proteins, bacterial products, and inflammatory mediators [136,142,169,223]. Human studies have yet to assess the full sequence from a defined metabolic exposure through a measured seminal mediator to subsequent sperm dysfunction within a longitudinal design.

Third, direct evidence for metabolically driven post-testicular epigenetic changes remains limited. Human studies report sperm DNA methylation differences associated with obesity or bariatric surgery [161,162]. Animal studies document epididymal RNA transfer and embryonic gene-expression effects [112,163,164,165], altered epididymal EV protein cargo in obese mice [111], and diet-related sperm mitochondrial RNAs linked to offspring metabolic phenotypes [166]. These separate findings leave the metabolic exposure–EV RNA–sperm–offspring pathway incompletely tested. Sperm DNA fragmentation may arise during spermatogenesis, when chromatin remodeling and protamine packaging affect nuclear integrity; during epididymal maturation, when oxidative DNA lesions may accumulate; and after ejaculation, when seminal ROS can cause further damage [124,129]. In mature human sperm, OGG1-mediated processing of 8-OHdG and limited downstream base-excision repair may leave abasic intermediates that precede DNA strand breaks [157,158].

Fourth, the low microbial biomass of semen makes sequencing results sensitive to background DNA from collection materials, reagents, and adjacent genital sites [66,68,70,71]. Relative-abundance data offer limited information on absolute microbial load and viability. Negative controls, absolute bacterial quantification, and longitudinal paired-partner sampling are needed to assess microbial associations with metabolic and reproductive outcomes [71,118].

Finally, the clinical evidence for antioxidant supplementation varies across compounds, doses, treatment durations, and study populations. A Cochrane review of 90 randomized trials rated the evidence for improved live birth as very low certainty; the apparent benefit was no longer evident after excluding studies at high risk of bias [224]. In the multicenter MOXI trial involving 174 men, a combined antioxidant formulation did not improve sperm motility, morphology, DNA fragmentation, or cumulative live birth [225]. The 2025 SUMMER trial involving 1171 men also found similar ongoing pregnancy rates with supplementation and placebo (33.8% versus 37.5%; adjusted odds ratio = 0.85, 95% CI 0.66–1.09) [226]. These trials enrolled heterogeneous fertility-care populations without selection based on measured seminal oxidative stress. Physiological ROS participate in capacitation and acrosomal signaling [45,46,47], and a recent narrative review discussed reductive stress as a potential consequence of excessive antioxidant exposure [227]. Compound-specific dosing, baseline redox status, and clinical pregnancy or live-birth outcomes require evaluation in adequately powered trials.

### 7.4. Priorities for Future Research

Future studies should prioritize longitudinal paired systemic–seminal–sperm studies that establish temporal sequence, together with causal mediation studies that test whether specific seminal factors transmit metabolic injury to sperm. Further priorities include analyses of metabolically driven epididymosomal RNA and protein cargo transfer and contamination-controlled seminal microbiome studies using absolute quantification to distinguish viable organisms from background signals. These priorities would move the field from association toward testable causal models and clinically actionable intervention targets.

## 8. Conclusions

The seminal microenvironment provides a useful framework for linking systemic metabolic disease with post-testicular sperm injury. Current evidence indicates that obesity, insulin resistance, dyslipidemia, inflammation, altered lipid metabolism, redox imbalance, and microbial signals may reshape seminal plasma and converge on sperm membranes, mitochondria, motility, capacitation, DNA integrity, and potentially epigenetic information. This model extends the metabolic–reproductive axis beyond the testis and highlights a tractable preconception interface. However, the field still requires longitudinal and mechanistic studies that connect systemic metabolic status, defined seminal changes, sperm outcomes, and fertility endpoints in the same individuals. Metabolic correction, antioxidant support, and microbiome-targeted approaches remain promising, but their benefits should be tested against clearly specified seminal targets. Recognizing the seminal microenvironment as an active and modifiable compartment may help guide future research and improve mechanism-directed strategies for protecting male reproductive function in metabolic disease.

## Figures and Tables

**Table 1:** Evidence categories, study types, and reported findings for components of the seminal microenvironment.

Evidence Category	Seminal Component	Metabolic Context	Reported Findings	Evidence Type	Ref.
Metabolic status and seminal measures	Leukocytes	Elevated BMI	In 382 men, BMI correlated with seminal leukocyte count (ρ = 0.112, *p* = 0.030).	Human cross-sectional study	[75]
Metabolic status and seminal inflammation	Inflammatory cytokines	Metabolic syndrome	Seminal IL-6, IL-8 and TNF-α profiles varied among metabolic-syndrome cohorts.	Human case–control and cross-sectional studies	[81,83]
Metabolic status and glandular inflammation	Accessory glands	HFD-induced obesity	HFD was associated with NF-κB/STAT3-related inflammation in mouse prostate and seminal-vesicle tissues.	Mouse in vivo studies	[84,85]
Obesity and epididymal EV cargo	Epididymal extracellular vesicles	HFD-induced obesity	Epididymal EV protein cargo differed in obese mice under oxidative and endoplasmic-reticulum stress.	Mouse in vivo study	[111]
Metabolic status and seminal metabolites	Seminal metabolome	Obesity	Obese men with sperm abnormalities had higher seminal-plasma spermidine and spermine.	Human cross-sectional studies	[89,119]
Seminal metabolites and sperm lipids	Lipid/fatty-acid axis	Elevated BMI	Seminal-plasma L-carnitine correlated with sperm fatty acids; separate cohorts reported BMI-related changes in sperm lipids.	Human cross-sectional studies	[90,95,120]
Physiological role of seminal fructose	Fructose	Metabolic and androgenic regulation	Seminal-vesicle-derived fructose is a substrate for sperm energy metabolism after ejaculation.	Human studies and narrative reviews	[34,38,121]
Seminal trace elements and sperm quality	Zinc and selenium	Fertility-related cohorts	A meta-analysis found lower seminal-plasma zinc in infertile men; seminal selenoprotein *p* was associated with sperm quality.	Systematic reviews, meta-analyses and human studies	[104,105,106,107,108,109,110]
Metabolic status and seminal microbiota	Seminal microbiome	HFD/obesity	HFD altered mouse seminal microbiota; human seminal microbial profiles were associated with BMI or sperm endpoints.	Mouse in vivo and human observational studies	[114,115,116]

Abbreviations: BMI, body mass index; EV, extracellular vesicle; HFD, high-fat diet; IL-6, interleukin-6; IL-8, interleukin-8; NF-κB, nuclear factor κB; STAT3, signal transducer and activator of transcription 3; TNF-α, tumor necrosis factor α.

**Table 2:** Mechanistically supported pathways linking alterations in the seminal microenvironment to sperm dysfunction.

Category	Seminal Insult or Altered Factor	Mechanistic Pathway	Main Target in Sperm Cells	Effect on Sperm Quality	Refs.
Oxidative–inflammatory	Activated seminal leukocytes	Excess extracellular ROS, cytokines, and proteases induce oxidative bystander injury	Membrane, mitochondria, and DNA	Lipid peroxidation, impaired motility and increased DNA damage	[122,123,124,125,126,127,128,129]
Oxidative–inflammatory	Pro-inflammatory cytokines, particularly TNF-α	Mitochondrial depolarization, nitric-oxide-related stress, and apoptotic signaling	Mitochondria and apoptotic machinery	Reduced motility, mitochondrial dysfunction, and apoptosis	[130,131,133,134,135,136,137,187,188]
Lipid remodeling	Altered cholesterol and fatty-acid levels	Disturbed membrane lipid composition, fluidity, and cholesterol dynamics, interfering with capacitation-related membrane remodeling	Plasma membrane	Impaired capacitation and acrosome reaction	[90,120,139,140,141,142,143,144]
Lipid peroxidation	Lipid-peroxidation-derived 4-HNE	Electrophilic adduction of succinate dehydrogenase and motility- and capacitation-related proteins, with the amplification of mitochondrial electron leakage and secondary ROS production	Mitochondria; flagellar and capacitation-related proteins	Impaired motility and capacitation, mitochondrial dysfunction, apoptosis, and oxidative DNA damage	[148,149,152,153,154]
Microbial	Bacterial adhesion and agglutination	Direct sperm–bacterium interaction and aggregation	Sperm surface and flagellum	Restricted progressive motility; increased focal microbial exposure	[167,168]
Microbial	Soluble bacterial products	Mitochondrial depolarization, phosphatidylserine redistribution, and membrane oxidative injury	Mitochondria and plasma membrane	Reduced motility, viability, and membrane integrity	[169,185]
Microbial	Bacterial lipopolysaccharide	Activation of sperm Toll-like receptor 2 and Toll-like receptor 4, triggering oxidative and apoptotic signaling	Sperm innate-immune and acrosomal signaling machinery	Oxidative stress, apoptosis, and abnormal acrosome reaction	[170,171]
Microbial	Biofilm formation and persistence	Prolonged bacterial viability and sustained inflammatory exposure	Seminal milieu and sperm environment	Persistent oxidative and inflammatory injury	[172,173]

Abbreviations: 4-HNE, 4-hydroxynonenal; DNA, deoxyribonucleic acid; ROS, reactive oxygen species; TNF-α, tumor necrosis factor-α.

**Table 3:** Human intervention studies reporting metabolic, seminal, and sperm-related outcomes.

Intervention	Seminal or Related Measurement	Sperm or Reproductive Endpoint	Evidence Type	Refs.
Diet-induced weight loss	BMI/systemic metabolic measures	Sperm concentration and total count	Human randomized trial	[189]
Lifestyle weight-loss program	BMI reduction	Sperm DNA fragmentation	Human intervention cohort	[190]
Structured exercise	Seminal IL-6, TNF-α, ROS, MDA, antioxidant measures	Semen quality and sperm DNA fragmentation	Human randomized trial	[194]
Metabolic/bariatric surgery	Seminal IL-8 or lipid-peroxidation measurements	Sperm motility, vitality, mitochondrial activity, and DNA integrity	Human prospective studies	[156,195]
N-acetylcysteine, 600 mg/day for 3 months	Seminal total antioxidant capacity and MDA	Sperm count, motility, morphology, and DNA fragmentation	Human intervention trial	[198]
Coenzyme Q10	Seminal CoQ10, antioxidant enzymes, oxidative indices	Seminal biochemical endpoints	Human double-blind randomized trial	[199]
DHA with vitamin E	Seminal oxidative stress; sperm phospholipids	Semen parameters	Human randomized trial	[200]
Synbiotic (FamiLact)	Sperm chromatin and protamine-related indices	Sperm concentration, motility, morphology, DNA integrity	Human triple-blind trial	[203]

Abbreviations: BMI, body mass index; CoQ10, coenzyme Q10; DHA, docosahexaenoic acid; IL-6, interleukin-6; IL-8, interleukin-8; MDA, malondialdehyde; ROS, reactive oxygen species; TNF-α, tumor necrosis factor-α.

**Table 4:** Distinct metabolic phenotypes and the measured reproductive evidence relevant to the seminal microenvironment.

Metabolic Phenotype	Defining Systemic Features	Reported Findings	Refs.
Obesity/elevated adiposity	High BMI/central adiposity; adipose inflammation and frequently concurrent insulin resistance.	Human cross-sectional studies reported seminal-plasma polyamine differences and variation in seminal microbiota in relation to semen parameters and sperm DNA fragmentation. An in vivo study in obese mice found changes in epididymal EV protein cargo.	[89,111,115,119]
Insulin resistance	Reduced insulin sensitivity; elevated fasting insulin or HOMA-IR, with variable glycemia.	In a human observational study of 792 men with idiopathic infertility, higher HOMA-IR was associated with SDF ≥ 30% (adjusted OR = 1.8).	[210]
Type 2 diabetes mellitus	Diagnosed chronic hyperglycaemia, commonly with insulin resistance.	A retrospective matched cohort comparing 106 men with T2DM and 212 controls reported lower semen volume and progressive sperm motility, with similar fertilization, implantation and pregnancy outcomes.	[211]
Dyslipidemia	Hypercholesterolemia or other changes in serum TG, TC, LDL-C and HDL-C.	An observational study of 631 subfertile men found a weak serum–seminal TG correlation (r = 0.081) and associations between seminal lipids and selected semen parameters. In vitro experiments on human sperm reported altered cholesterol/sphingolipid signaling, oxidative indices and capacitation.	[212,213]
Metabolic syndrome	Clustering of adiposity, dysglycaemia, dyslipidemia and increased blood pressure.	Human case–control and cross-sectional studies reported differing seminal inflammatory cytokine profiles: one cohort detected higher concentrations, whereas another found a comparable cytokine network, with sperm-related outcomes also assessed.	[81,83]

Abbreviations: BMI, body mass index; EV, extracellular vesicle; HOMA-IR, homeostatic model assessment of insulin resistance; OR, odds ratio; SDF, sperm DNA fragmentation; T2DM, type 2 diabetes mellitus; TG, triglycerides; TC, total cholesterol; LDL-C, low-density lipoprotein cholesterol; HDL-C, high-density lipoprotein cholesterol.

## Data Availability

No new data were created or analyzed in this study. Data sharing is not applicable to this article.

## Abbreviations

The following abbreviations are used in this manuscript:

4-HNE	4-hydroxynonenal
16S rRNA	16S ribosomal RNA
BMI	body mass index
DHA	docosahexaenoic acid
HFD	high-fat diet
IL-6	interleukin-6
IL-8	interleukin-8
NADPH	reduced nicotinamide adenine dinucleotide phosphate
NF-κB	nuclear factor kappa B
Nrf2	nuclear factor erythroid 2-related factor 2
PRDX6	peroxiredoxin 6
PUFAs	polyunsaturated fatty acids
ROS	reactive oxygen species
SMD	standardized mean difference
TNF-α	tumor necrosis factor-α
